# Transfer of anatomy during surgical clerkships: an exploratory study of a student-staff partnership

**DOI:** 10.5116/ijme.62eb.850a

**Published:** 2022-08-31

**Authors:** Josefin Ivarson, André Hermansson, Björn Meister, Hugo Zeberg, Klara Bolander Laksov, Wilhelmina Ekström

**Affiliations:** 1 Department of Clinical Neuroscience, Karolinska Institutet, Stockholm, Sweden; 2 Department of Neuroscience, Karolinska Institutet, Stockholm, Sweden; 3 Centre for Psychiatry Research, Department of Clinical Neuroscience, CAP Research Centre, Karolinska Institutet, Sweden; 4 Department of Molecular Medicine and Surgery, Karolinska Institutet, Stockholm, Sweden

**Keywords:** Vertical integration, clinical anatomy, undergraduate medical education, near-peer learning, student-staff partnership

## Abstract

**Objectives:**

This qualitative study aims to explore how fourth-year medical students on the surgery course perceived a clinical anatomy workshop organised by near-peer student teachers in partnership with faculty.

**Methods:**

Forty-seven medical students participated in a workshop on clinical anatomy in the dissection laboratory. A voluntary response sampling method was used. The students' perceptions of the workshop were explored through a thematic content analysis of transcribed, semi-structured group interviews and written comments.

**Results:**

A majority of the students had not revisited the dissection laboratory since their second year, and all students described the workshop as a unique opportunity to vertically integrate anatomical knowledge. Four main themes were identified as most valuable for the students' learning experience, namely that the workshop 1) was taught by knowledgeable and friendly near-peer tutors (NPTs), 2) consisted of highly relevant anatomical content, 3) offered a hands-on experience of cadavers in the dissection laboratory, and 4) was taught in a focused session in the middle of the surgery course.

**Conclusions:**

This study shows how hands-on workshops in clinical anatomy, developed in student-staff partnerships and taught by NPTs, can enable senior medical students to recall and vertically integrate anatomical knowledge during surgical clerkships. The results have implications for curriculum design, giving voice to senior students’ wishes for spaced repetition and vertical integration of pre-clinical anatomy knowledge during their clinical training. Moreover, this study may inspire other students and faculty to develop similar near-peer teaching activities through student-staff partnerships.

## Introduction

A well-known challenge of teaching medical curricula is how to help students remember and apply their pre-clinical knowledge in clinical contexts, such as their anatomical knowledge. Inadequate levels of anatomical knowledge amongst senior medical school students have consistently been reported by clinical teachers,^1^^, ^^2^ junior doctors,^3^^, ^^4^ and by medical students themselves.^5^^-^^9^ In one cohort, only 14% of fourth-year medical students, reported feeling confident in their anatomical knowledge.^9^ One way this problem has been understood is in terms of the well-known difficulties of transferring knowledge from one context to another.^10^ For example, students may know anatomy well in one context, such as on human cadavers in the dissection laboratory, but they may experience difficulties applying this knowledge in a clinical setting,^8^^,^^11^ such as examining a patient in the emergency department. Many medical schools, therefore, strive to integrate the basic and clinical sciences during the early years of undergraduate medical education by teaching anatomical structures in relation to common clinical concepts and skills.^12^^-^^14^ Such vertical integration is an efficient way to facilitate knowledge transfer from the pre-clinical to the clinical context.^15^

However, a curriculum design that integrates basic sciences and clinical anatomy in the early years of medical education may not be sufficient to maintain medical students’ anatomical knowledge in later years.^16^^-^^18^ One reason for this might be the often significant time gap between when they are first taught anatomy and when they are expected to apply it clinically several years later. Moreover, the limited hands-on clinical experience of medical students in their early years may hamper clinical integration of the basic sciences at this stage of their education. Studies have also found that clinical teachers may lack educational strategies to overcome the problem of transfer,^19^ and that even when clinical teachers think they are teaching basic anatomy during clinical rotations, students may not experience this to be the case.^11^

There is a growing number of published examples of learning activities designed to enable senior medical students to vertically integrate their anatomical knowledge in the latter part of their studies.^20^^-^^31^ In general, these activities show high student satisfaction and a marked improvement in anatomical knowledge. These interventions vary in three respects: 1) some address core curricular content in undergraduate medical education, while others are electives bordering on postgraduate training in surgery; 2) some are several weeks long, while others just a few hours; and 3) some happen during summer holidays, while others are nested within regular clinical rotations. Three features that are common to most of these interventions are small-group discussions with teachers, a case-based format, and some hands-on activities such as prosections or dissections of cadavers, or building plastic anatomical structures. The majority of these interventions are initiated, developed and taught by faculty – either surgical faculty alone or as interdisciplinary collaborations between clinical and anatomical members of faculty.

There is a strong tradition of using near-peer students (NPTs) as teachers of anatomy in undergraduate medical education, with benefits both for the students and the student teachers.^32^^-^^40^ NPTs are students on the same curriculum as the learners but at a more advanced stage – usually a year or two ahead.^39^ Having recently gone through the same courses, NPTs understand the core learning outcomes the students have to master, as well as how to explain the areas that might be especially difficult for students to grasp. Teaching anatomy to junior medical students is also an effective way for senior student teachers to improve their own anatomical knowledge.^31^^,^^41^^,^^42^ Near-peer teaching programmes can offer student teachers continuous vertical integration of anatomical knowledge as they revisit and teach basic sciences while advancing in the clinical curriculum.

However, in nearly all studies on near-peer learning of anatomy, the learners are in their pre-clinical years of study.^31^^,^^42^ As rare exceptions to this, Hall and colleagues^35^ describe a neuroanatomy refresher course taught by fourth- and final-year medical students to third-year medical students on clinical rotations, and Morris and colleagues^43^ describe how third- to fifth-year clinical students gained confidence from a one-day anatomy workshop taught by NPTs. The neuroanatomy course reported by Hall and colleagues^35^ was not just taught by senior medical students but also initiated by them, developing the content in collaboration with the neuroanatomy course leader. This is rather unusual, as not much is published in terms of the roles students could and do play in initiating and developing courses.^44^^,^^45^ Within the broader literature on teaching and learning in higher education, the notion of students as partners in higher education is gaining ground.^46^^-^^48^ Student-staff partnerships in pedagogical planning processes have been shown to have the potential to enhance the engagement, motivation and enthusiasm around the learning process for both students and staff.^49^^, ^^50^

In summary, several researchers have called for more studies on initiatives that allow senior medical students to vertically integrate anatomical knowledge during their clinical rotations, i.e., revisit basic science anatomy in relation to the clinical issues they are facing.^3^^,^^9^^,^^11^^,^^19^^,^^51^^-^^53^ There is also limited knowledge about the potential of using senior NPTs to help other senior medical students revisit basic anatomical knowledge during their clinical clerkships. Lastly, there is a lack of published examples of student-staff partnerships to enhance vertical integration of anatomy amongst senior medical students.

The aim of this qualitative study is to explore senior medical students’ perceptions of a clinical anatomy workshop initiated by students, developed in a student-staff partnership, and taught by NPTs. Specifically, the research objectives are to explore what senior medical students perceived to be the most valuable features of the workshop and why. This study thereby seeks to understand student perspectives on how vertical integration of anatomical and surgical knowledge can be facilitated. It also aims to provide a concrete example of how to build a workshop using NPTs in close partnership with faculty.

## Methods

### Study design and participants

An explorative, qualitative study was done, and the data were analysed using thematic content analysis within a social constructivist paradigm.^54^ Participants were selected using voluntary response sampling. Participation in both the workshop and data collection for the study was voluntary.

Two identical workshops were held in order to accommodate a larger number of participants. In total, 47 fourth-year medical students participated in the workshops, which were held on two consecutive Saturdays in the middle of the surgical course in spring 2019. Of these, 44 students volunteered to participate in the study. Out of the three students who did not participate in the study, one had to leave the workshop because of nausea, one had to leave early because of other commitments, and one gave no reason for not participating in the study.

Half of the students who participated in the study were male, and half were female. In terms of age, 70% were between 21 to 26, and 30% were between 27 and 45, which corresponds to the overall distribution of age and sex in the medical programme. Students had completed different clinical clerkships prior to the workshop. For example, some had already done their orthopaedic clerkships, whereas others were scheduled to have theirs later in the semester. Of the six mandatory surgical clerkships on the course, the students had completed two or three at the time of the workshop. The following percentages of students reported having completed these clerkships: general surgery, 20 students (43%); vascular surgery, 18 students (38%); urology, 27 students (57%); orthopaedics, 15 students (32%); anaesthesiology, 20 students (43%); breast and endocrine surgery, 14 students (30%).

Ethical approval for the study was obtained from the Regional Ethical Review Board in Stockholm for the Department of Molecular Medicine and Surgery. Informed consent was obtained in writing from each participant.

### Data collection methods

In order to explore in-depth the participants’ perceptions of the workshop, semi-structured group interviews were carried out and written comments were collected. After each workshop, the students were divided into groups of 2-7 students. First, they were given time to write down their reactions to the workshop. Next, one NPT per group conducted a semi-structured interview of their group according to an interview guide developed for this study. The NPTs were instructed to follow the interview guide and add open-ended questions to prompt elaboration. In total, 10 group interviews were conducted, five after each workshop. The interviews lasted an average of 21 minutes, were audio recorded and later transcribed verbatim. The interviews were carried out in Swedish. Quotes from the interviews referred to in the findings section have been translated into English.

### Data analysis

In line with the explorative character of the study, two of the authors (AH and JI) each carried out a thematic content analysis^54^ of the transcribed group interviews and written comments through iterative readings. Their analyses were compared and discussed, first between AH and JI and then in meetings with the rest of the research team – all of whom had read the transcribed interviews individually. This process was then carried out again iteratively in parallel with an extension of the literature review. To increase the trustworthiness of the analysis, the findings from the written comments were compared with the findings from the group interviews, but no major contrasts were identified. An external researcher (KBL) with experience in qualitative educational research was involved in the project to increase credibility.

### Setting

The study was conducted at Karolinska Institutet, where the undergraduate programme in medicine comprises five and a half years of study. The curriculum is explicitly designed with the intention of facilitating the vertical integration of knowledge throughout the programme. The first two years are mainly oriented toward pre-clinical studies on campus, followed by three years of clinical courses in a healthcare setting, punctuated by a semester devoted to writing a master’s thesis. Approximately 160 students are enrolled each semester.

The basic science anatomy curriculum spans the first three semesters. During the second and third semesters, students spend four weeks studying the musculoskeletal system and four weeks studying the topographical anatomy of the head, neck, thorax, abdomen and pelvis, as well as neuroanatomy. In addition to lectures, seminars and self-study, a significant amount of time is dedicated to near-peer-led group dissections of embalmed human cadavers.

After the abovementioned pre-clinical courses, the majority of students never return to the dissection laboratory. However, about a quarter of them return to the dissection laboratory as part of a well-established option for NPTs. Upon entering their fourth semester, students can apply to become a NPT in anatomy, where their main responsibility will be to guide groups of second- and third-semester students as they dissect a cadaver according to a local manual. After doing this for two semesters, NPTs can apply to become senior NPTs, whose responsibility it is to oversee the dissections in the laboratory with support from faculty. In total, there are about 20 senior NPTs at any given time, who naturally tend to develop a broader and deeper understanding of anatomy when compared with their peers.

The surgical clerkship takes place during the fourth year of study and runs over 18 weeks, with students being placed at one of four different hospital sites. To get experience in different subspecialties, students participate in clinical clerkships that change every other week according to a rotating schedule. In parallel with the clerkships, there are lectures and seminars held at each hospital site. By the time they enter their surgical clerkships, it is more than one and a half years since the students last studied anatomy, except for the NPTs and senior NPTs.

### Developing the workshop

This initiative came about when two of the authors, AH and JI, identified an opportunity for fourth-year medical students to review and deepen their anatomical knowledge. At the time, AH and JI were medical students who had just completed the surgical course, had experience as senior NPTs in the dissection laboratory, and were authors of the local dissection manual. A workgroup was formed, consisting of AH, JI and faculty from the anatomical and surgical departments. A pilot workshop was arranged during autumn 2018, where feedback from students and NPTs was collected and refinements were made before the workshop proper in spring 2019. During the development of the workshop, input was sought from surgical and anatomical faculty, as well as medical students in their surgical rotations, regarding the areas or concepts they considered particularly valuable to revisit during the workshop. Curriculum mapping was done in order to identify learning outcomes that were suitable for the dissection laboratory.

Six embalmed cadavers were used during the workshop. All bodies were voluntarily donated through the local donation programme for educational purposes. All donors gave signed consent prior to death, were above the age of 70, and were deemed suitable for dissection (e.g., they had no heavy tumour burden or major trauma). The workshop was a pilot project to be integrated into the pre-existing medical undergraduate curriculum at Karolinska Institutet in Sweden.

The workshop lasted four hours, consisted of six stations, and was designed for approximately 30 students to be divided into groups of 4-6 students. The groups rotated through each station every 20 minutes. At each station, the students gathered around a pre-dissected cadaver and were guided by a senior NPT. Each station started with a clinical case presentation and a review of the main learning outcomes in relation to the surgical course curriculum. This was followed by a detailed exploration of the anatomical structures on the cadaver, and the students were given time to explore the cadaver and ask questions. The senior NPTs gave short summaries of the main points and asked the students questions in relation to the learning outcomes.  The students were not expected to do any preparation before the workshop, but the senior NPTs received written instructions regarding their stations a month before. Most of the NPTs also attended an evening session a week before the workshop in order to familiarise themselves with the cadavers and their station’s learning outcomes. At this session, the NPTs were encouraged to perform dissection of their cadavers for better exposure of relevant anatomical structures. On the day of the workshop, the senior NPTs gathered two hours prior to the arrival of the students and were reminded by AH, JI and WE of the setup and purpose of the workshop, and were given time to prepare and familiarise themselves with the cadavers.

## Results

### The students’ overall perceptions of the workshop

All students expressed great enthusiasm about the workshop, saying it was an effective, fun and valuable component of their learning experience during their surgical clerkships.

A majority of the students had not revisited the dissection laboratory since their third semester, and many of them expressed their appreciation that the workshop addressed their low levels of anatomical knowledge, for example:

“I had forgotten almost all anatomy when I started the seventh semester. It was like, ‘oh crap, now it’s time for my clerkship in orthopaedics – but I’ve forgotten almost everything.’” (No. 7, Male)

Nearly all the students attributed their low level of anatomical knowledge to the long gap between their basic anatomy courses and their surgical clerkships and the lack of any structured repetition in between. A handful of them had been anatomy tutors and/or rated their anatomical knowledge prior to the workshop as ‘good’, but they too expressed a strong need to bring this knowledge “to the forefront again, so it lies closer at hand” (No. 30, Female).

When asked about suggestions for improvements to the workshop, the students wanted it to last longer, to include more clinical cases, to be a mandatory part of the surgical course, and to be offered in an adapted format during clerkships in internal medicine. They also wanted written course material so they could prepare in advance and revise afterwards. Several students said that the workshop was not only valuable in and of itself, but that it motivated them to study more anatomy during the rest of their surgical clerkships. They felt enthused by the workshop and it served to highlight the areas they needed to revise further:

“I thought it was a pretty motivational workshop. It feels like it’s going to be fun to understand anatomy now in order to understand the rest of the surgical course, too.” (No. 2, Female)

“I thought the whole thing became a bit like a quiz, where I could notice what I didn’t know, what others were very good at, and what I was not good at. […] Now, I understand what parts I haven’t studied enough.” (No. 11, Male)

### What students perceived to be the most valuable features of the workshop and why

Four main themes were identified in terms of what the students perceived to be most valuable for their learning experience, namely that the workshop 1) was taught by knowledgeable and friendly NPTs, 2) consisted of highly relevant anatomical content, 3) offered hands-on experience of cadaver specimens in the dissection laboratory, and 4) was taught in a focused session in the middle of the surgical clerkships.

### Knowledgeable and friendly NPTs

A central theme in the students’ perceptions of the workshop was their enthusiasm about the NPTs, i.e., how knowledgeable and well-prepared they were and the friendly atmosphere they created. Aside from being knowledgeable about anatomy, the students described the NPTs as knowledgeable in relation to their learning needs: “[the NPTs] know what’s important to know, what’s good to know, and what’s hard to know” (No. 22, Male). Moreover, they thought the NPTs created a friendly atmosphere where “no question felt stupid, it was very relaxed – a good learning environment” (No. 40, Female).

Finally, they valued the interactive format in small groups, where the NPTs invited them “to be active and involved” in a way “that made the anatomical content stick” (No. 39, Male).

### Relevant anatomical content

The students also valued the highly relevant anatomical content – a theme that was divided into three sub-themes. First, the students appreciated the focus on anatomical regions they found particularly hard to master, such as the inguinal region and the retroperitoneal space. Secondly, they found it useful that the content of the workshop was framed using concrete clinical cases. They explained that beginning each station in the workshop with a clinical vignette made “it easy to understand the clinical relevance” (No. 14, Female).  Thirdly, the students valued the clinical cases being clearly linked to core curricular learning outcomes for their surgical clerkships. They found the focus on the core curriculum especially useful, partly because “in the wards, it’s not always like one’s supervisor knows exactly what one’s intended learning outcomes are” (No. 1, Female), and partly because of the arbitrariness of the anatomical content they happened to encounter during their clerkships:

“It’s a bit random – if you’re lucky, you get in on a hernia [surgery], but if you’re not, then how are you supposed to learn? Sitting at home, reading a book? That’s really hard.” (No. 30, Female)

One student felt that the focus on the core curriculum made “…learning somehow fairer, giving everybody the chance to go through the basics of complicated stuff, like hernias” (No. 5, Male). For some students, the workshop did not just highlight the core anatomical curriculum but also the core learning outcomes in the surgical course in general:

“Now, I feel that I really understand what I’m supposed to understand during the surgical course.” (No. 3, Male)

### Hands-on experience in the dissection laboratory

The students found the hands-on experience of seeing and touching anatomical structures on the pre-dissected cadavers valuable:

“It’s about getting another dimension to the whole surgical course. Sometimes, we’re at clinical skills training centres, then we have lectures, then we’re in the wards, watching surgeries and maybe we get to touch something but, in an integrated session like this, where one can both touch and look around a bit, it becomes 4D – to be able to touch.” (No. 9, Male)

Another student, continuing the conversation:

“I really agree, it was like connecting theory and practice. Now I can actually understand why it’s crowded there and there, because I can see and feel it, you know. Not just visualise it when I read about it.” (No. 13, Female)

The students elaborated on why this feature was so valuable by comparing it with some perceived difficulties of learning anatomy in the clinical learning environment:   

“Here [in the dissection laboratory], one can flip through layers of the body and understand… I was at a hernia surgery the other week, and then you go straight in. The thing is, during surgery, there’s this little incision to look through, whilst here we get to see the whole body opened and then we understand a bit more what structures are actually there. The anatomy sticks better that way.” (No. 21, Female).

### A focused anatomy learning activity in the middle of the surgical course

Finally, the students described why they valued having an anatomy workshop timed to the middle of their surgical clerkship. First of all, they appreciated having a session dedicated to clinical anatomy, away from the often stressful clinical setting:

“It’s a bit calmer [here in the workshop], and you can actually stop and ask, ‘hey, can we check that out, can we look at the kidneys?’ In the middle of a surgery, you can’t really say, ‘hey, can everybody stop because I want to look at that…’  It doesn’t work that way [laughter].” (No. 42, Male)

“During surgeries, everything happens so quickly. And you can’t ask too many questions because, well, they have to work, you know.” (No. 30, Female)

Moreover, the students also appreciated the extended focus on anatomy in the workshop, in contrast to what some experienced as too little focus on it during the lectures for the clinical clerkships:

“In the lectures by the orthopaedic surgeons, they usually start by quickly going through like, ‘this is the anatomy of the foot’. Bam, bam, bam! And then they turn straight to the injuries.” (No. 5, Female)

Some students highlighted how, occasionally, clinical teachers and lecturers did allocate sufficient time to revising basic anatomy, which they appreciated.

Regarding the timing of the workshop to the middle of the surgical course, the students spontaneously discussed different potential timings of the workshop, i.e., during the first week of the course, in the middle of the semester, or towards the end, closer to the exam. The general consensus was that the best time was in the middle of the semester, during the surgical clerkships:

“[Having it in the middle of the semester] made it more exciting because some stuff was totally new, so then one was like, ‘oh how exciting it will be to do the orthopaedic clerkship’, whereas some stuff was repetition, like the hernia station where one could take a step back and be like ‘now, really, what kind of a structure is this...’” (No. 9, Male)

“In one way, I would have liked to have this prior to all my clinical clerkships, but at the same time, I almost learned the most from those stations where I had already had the relevant clerkship because then I didn’t have so many questions but just a few things I wanted to double check and straighten out.” (No. 10, Female)

The students also described how the timing of the workshop to the middle of clerkships gave anatomy a whole new salience for them. Two students remarked:

“It’s invaluable to be able to return to the dissection laboratory and take on anatomy again with a clinical perspective when one has more to anchor that knowledge in.” (No. 21, Female)

“One has a completely different context to relate one’s knowledge to now compared with the first year.  It’s great to study it [anatomy] then, but you don’t always get – in the same way – how it is clinically relevant. So it’s great that it’s in conjunction with the surgical clerkship one gets to repeat.” (No. 36, Male)

The students commented on how, even though the anatomy taught in the early years was clinically relevant, it was not until they had their own clinical experience that they really understood the clinical relevance:

“Before the start of the surgical clerkship, I looked over the lecture notes from [a lecturer in anatomy in the second semester], and at the time, I was not aware of this, but now, looking through it again, it’s very relevant anatomy – it’s really stuff one needs to know.” (No. 38, Female)

## Discussion

The aim of this study was to explore senior medical students’ perceptions of a clinical anatomy workshop initiated by students, developed in a student-staff partnership, and taught by NPTs. Overall, the students were enthusiastic about it and thought it was a valuable way to improve their anatomical knowledge and motivate them to continue revising anatomy. Many students wished for more such learning activities in the future and proposed that the workshop be incorporated into the core curriculum of the surgical course and the internal medicine course. This positive overall perception is consistent with findings from similar studies.^21^^-^^31^

### The need for vertical integration of anatomical knowledge during the clinical years

Echoing previous research, the students in this study said they valued the workshop because they felt their anatomical knowledge was inadequate.^9^^,^^11^^,^^53^ They partly attributed this problem to the fact that there had been no structured spaced repetition of anatomy since their first three semesters. They expressed a wish for a spiral curriculum that could allow them to revisit anatomy in a systematic manner as they progressed through medical school – especially during their clinical clerkships – which is something long advocated for in medical education research.^13^^,^^20^^,^^55^ The timing of the workshop was seen as crucial, because even if the students had been taught anatomy during preclinical semesters, it was only later, during their clerkships, that they had an experiential understanding of the clinical relevance and could vertically integrate the knowledge with surgical patient care.

The students described how barriers to revising and learning anatomy in the clinical learning environment made them value the workshop in four ways. First, they felt many clinical supervisors lacked awareness of their core curricular outcomes, leaving students with a sense of ‘navigating without a map’.^56^ The workshop provided them with such a map of anatomical knowledge that was relevant to the learning outcomes of the surgical course. Secondly, many students felt that lectures and surgeries happened so quickly that there was not enough time for properly learning anatomy. Indeed, Lazarus and colleagues assert that, even when clinical teachers report teaching anatomy within the overall context of clinical rounds, students seldom feel that it was really taught.^11^ Therefore, having workshops outside the everyday clinical setting may be an effective way to help students focus on anatomy. Thirdly, the students felt that the dissected bodies were a more useful learning modality compared to the anatomical structures in the operating theatres, because they allowed a better overview and an opportunity to flip through layers of tissue. This is consistent with previous studies that report exposure to dissections being strongly associated with medical students’ improved confidence level in anatomical knowledge.^55^^, ^^57^ In summary, only spending time in clinical surgery clerkships may not impart sufficient anatomical knowledge to medical students in the latter part of medical curricula.^28^^,^^58^ The results of this study reinforce and add nuance to calls for more vertical integration of anatomical knowledge during clinical years.^3^^,^^9^^,^^11^^,^^19^^,^^51^^-^^53^ Arranging learning activities where students can make explicit and purposeful connections between anatomical and clinical sciences at a micro-level (e.g., focusing on one case vignette and one part of a human cadaver at a time) enables knowledge integration as a cognitive activity within the learner.^59^

### Near-peer teaching of anatomy to senior medical students

The medical students in this study also described the NPTs as a key part of what made the workshop such a valuable learning experience. They found the NPTs to be knowledgeable, well-prepared, enthusiastic, approachable and friendly, which is consistent with the near-peer teaching literature in anatomy.^31^^-^^33^^,^^38^^,^^39^ The results of this study reveal that this near-peer teaching format is also a feasible option for senior medical students to teach other senior medical students anatomy relevant to surgical patient care. Previous studies of vertical integration report that senior medical students greatly value being taught clinical anatomy by faculty,^27^ expert clinicians^25^ and surgeons.^60^ In contrast, in this study, the NPTs were just one, two or three semesters ahead of the student learners. These findings thus widen the understanding of who can teach for vertical integration in the clinical years of medical school, i.e., NPTs who are close in the curriculum to the student learners. Indeed, a recent publication reports that senior medical students improved their knowledge of clinical surgery from near-peer taught sessions during the surgical clerkship.^61^

This study shows that much of the students’ positive perception of NPTs was due to their perceived approachability, i.e., the students felt comfortable asking them questions they might not have asked clinical supervisors. This common finding in NPT research is understood as a social congruence between the learner and the teacher.^31^^,^^62^^,^^63^ The students also indicated that the NPTs were able to teach important and difficult concepts in a way that simplified and facilitated their learning. This ability to teach at the right level for the learner is often attributed to a cognitive congruence between the teacher and learner.^62^^, ^^63^ In contrast to more senior teachers and faculty, students who have recently learned the course content may be better at recognising difficult concepts and barriers to learning from a student perspective, as well as conveying strategies for overcoming these.^31^^,^^63^

When it comes to learners’ self-perceived or measured knowledge gains after educational activities, NPTs are not inferior to more senior teachers, everything else being equal.^33^ However, the relative lack of anatomical and clinical knowledge may be a limitation for NPTs as substitutes for more experienced teachers.^31^ It is therefore possible that if the NPTs in this study were replaced by surgeons, the learning experience for the students might be the same or even better. In this context, this potential limitation may have been counteracted by recruiting a pool of particularly experienced and motivated NPTs. Indeed, representatives of other well-established student tutor programmes in anatomy stress the importance of securing good standards of anatomical and pedagogical skills amongst the NPTs.^64^

### Students as partners for increased vertical integration

This study is an example of how students, in collaboration with faculty, can initiate and develop a valuable learning activity for undergraduate medical education. The following aspects may have had particular relevance to the positive reception of this workshop. First, the workshop was conceived by two medical students who had just completed the surgical course, throughout which they had identified pertinent anatomical learning needs by talking to, and observing other students and clinical teachers. Secondly, these two students had recently developed new dissection manuals for the preclinical anatomical courses and were part of the anatomy tutor programme, so they knew what the pre-clinical students were learning and the concrete learning opportunities the cadavers could offer. This contextual familiarity helped them tailor the workshop to the needs of the students and the course, in line with essential aspects for the successful development of medical curricula.^65^ Moreover, by being part of the well-established near-peer tutor programme at Karolinska Institutet, the student authors had access to a network of senior tutors, which made collaboration easy and accessible. Likewise, by having a trustful relationship with both anatomical and surgical faculty before the workshop was initiated, the student authors were able to serve as a bridge, strengthening the relationship between the surgical and anatomical departments.

Such collaborations between pre-clinical and clinical faculty are an effective way to increase vertical integration.^66^^-^^68^ This collaborative approach seemed to increase the quality of the workshop in the implementation phase and contributed to the enthusiasm expressed by the participating students, NPTs and faculty. Finally, and crucially for successfully developing and implementing the workshop, key faculty members supported the initiative from start to finish, making the workshop possible from an organisational and financial standpoint. A recent qualitative research synthesis of students as partners in medical education highlighted the importance of continuing institutional and financial support for the longevity of similar initiatives and for students to contribute to student-staff partnerships^69^ – all of which applied in the setting presented in this article.

### Limitations

Participation in the workshop was voluntary, which means there was a self-selection bias that could have affected the students’ motivation and prior knowledge, but it is unclear what exactly this might entail for the current study. Also, having the NPTs conduct the interviews may have introduced a familiarity between the interviewees and interviewers from the workshop learning experience they had shared moments before. While this familiarity may have invited more honest feedback from students to NPTs, it may also have made students more prone to reporting only positive perceptions of the workshop and not feel comfortable voicing criticism. Moreover, the NPTs were not trained interviewers. In order to address these methodological issues, all the participating students were informed orally and in writing that their honest perceptions were needed to develop the workshop further. NPTs were given instructions on interviewing as well as an interview guide. The anonymous written comments were compared to the interview data to detect contrasting perceptions, though none were found.

## Conclusions

This exploratory study shows how hands-on workshops in clinical anatomy, developed in student-staff partnerships and taught by NPTs, can enable senior medical students to recall and vertically integrate anatomical knowledge during surgical clerkships. The results have implications for curriculum design, giving voice to senior students’ wishes for spaced repetition and vertical integration of knowledge during their clinical training. To enable such vertical integration, relevant

hands-on learning activities outside the clinical learning environment should be offered to senior medical students more often. This study may inspire other students and faculty to develop similar learning activities in student-staff partnerships using NPTs. Future studies could benefit from doing a longitudinal follow-up to assess whether the workshop affected students’ anatomical knowledge and learning during the rest of their surgical clerkships and beyond.

### Acknowledgments

The authors would like to thank all the participants in the study for their contributions. They would also like to express gratitude to the body donors’ invaluable contribution to medical education and to the NPTs for their help in preparing, facilitating and evaluating the workshops. This work was supported by Karin and Nils Rosander’s Fund and the Karolinska Institutet Educational Project Fund.

### Conflict of Interest

The authors declare that they have no conflict of interest.
